# Respiratory symptoms in children living near busy roads and their relationship to vehicular traffic: results of an Italian multicenter study (SIDRIA 2)

**DOI:** 10.1186/1476-069X-8-27

**Published:** 2009-06-18

**Authors:** Enrica Migliore, Giovanna Berti, Claudia Galassi, Neil Pearce, Francesco Forastiere, Roberto Calabrese, Lucio Armenio, Annibale Biggeri, Luigi Bisanti, Massimiliano Bugiani, Ennio Cadum, Elisabetta Chellini, Valerio Dell'Orco, Gabriele Giannella, Piersante Sestini, Giuseppe Corbo, Riccardo Pistelli, Giovanni Viegi, Giovannino Ciccone, SIDRIA-2 Collaborative Group

**Affiliations:** 1 Cancer Epidemiology Unit, AOU San Giovanni Battista Hospital – Center for Cancer Prevention (CPO Piedmont) and University of Turin, Via Santena 7, 10126 Turin, Italy; 2 Regional Environmental Protection Agency, Piedmont Region, Via Sabaudia 164, 10095 Grugliasco (Turin), Italy; 3 Centre for Public Health Research, Massey University Wellington Campus, PO Box 756 Wellington 6140 NZ; 4 Department of Biomedical Sciences and Human Oncology, University of Turin, Via Santena 7, 10126 Turin, Italy; 5 Department of Epidemiology, Rome/E Local Health Authority, Via di S. Costanza 53, 00198 Rome, Italy; 6 Department of Pediatrics, "Regina Margherita" Children's Hospital, University of Turin, P.zza Polonia 94, 10126 Turin, Italy; 7 I Pediatric Clinic, University of Bari, Piazza G. Cesare 11,70124 Bari, Italy; 8 Department of Statistics, University of Florence, Viale Morgagni 59, 50134 Florence, Italy; 9 Unit of Biostatistics, Istituto per lo Studio e la Prevenzione Oncologica (ISPO), Via San Salvi 12, 50135 Florence, Italy; 10 Epidemiology Unit, Local Health Authority, Corso Italia 19, 20122 Milan, Italy; 11 Unit of Pneumology and Allergology, Local Health Authority TO-2, Lungo Dora Savona 26, 10152 Turin, Italy; 12 Unit of Environmental and Occupational Epidemiology, Istituto per lo Studio e la Prevenzione Oncologica (ISPO), Via San Salvi 12, 50135 Florence, Italy; 13 Department of Prevention, Rome/G Local Health Authority – Tivoli Corso Garibaldi 7, 00034 Colleferro (Rome), Italy; 14 Unit of Preventive Medicine, Local Health Authority, Via Trento 6, 46100 Mantova, Italy; 15 Institute of Respiratory Diseases, University of Siena, Viale Bracci 3, 53100 Siena, Italy; 16 Department of Respiratory Physiology, Catholic University of Rome, Largo F.Vito 1, 00168 Rome, Italy; 17 CNR Institutes of Biomedicine and Molecular Immunology, Palermo, and of Clinical Physiology, Pisa. Via Ugo La Malfa 153, 90146 Palermo, Italy

## Abstract

**Background:**

Epidemiological studies have provided evidence that exposure to vehicular traffic increases the prevalence of respiratory symptoms and may exacerbate pre-existing asthma in children. Self-reported exposure to road traffic has been questioned as a reliable measurement of exposure to air pollutants. The aim of this study was to investigate whether there were specific effects of cars and trucks traffic on current asthma symptoms (i.e. wheezing) and cough or phlegm, and to examine the validity of self-reported traffic exposure.

**Methods:**

The survey was conducted in 2002 in 12 centers in Northern, Center and Southern Italy, different in size, climate, latitude and level of urbanization. Standardized questionnaires filled in by parents were used to collect information on health outcomes and exposure to traffic among 33,632 6–7 and 13–14 years old children and adolescents. Three questions on traffic exposure were asked: the traffic in the zone of residence, the frequency of truck and of car traffic in the street of residence. The presence of a possible response bias for the self-reported traffic was evaluated using external validation (comparison with measurements of traffic flow in the city of Turin) and internal validations (matching by census block, in the cities of Turin, Milan and Rome).

**Results:**

Overall traffic density was weakly associated with asthma symptoms but there was a stronger association with cough or phlegm (high traffic density OR = 1.24; 95% CI: 1.04, 1.49). Car and truck traffic were independently associated with cough or phlegm. The results of the external validation did not support the existence of a reporting bias for the observed associations, for all the self-reported traffic indicators examined. The internal validations showed that the observed association between traffic density in the zone of residence and respiratory symptoms did not appear to be explained by an over reporting of traffic by parents of symptomatic subjects.

**Conclusion:**

Children living in zones with intense traffic are at higher risk for respiratory effects. Since population characteristics are specific, the results of validation of studies on self-reported traffic exposure can not be generalized.

## Background

Vehicular traffic is a major source of outdoor air pollution. Several studies have reported associations between exposure to traffic pollutants in the zone of residence and increased frequency of respiratory tract illnesses [1-10]. The specific role of diesel exhaust from heavy traffic has been suggested in some of these studies [4,5,7], and airway inflammation due to exposure to diesel exhaust seems the likely biological mechanism [11,12]. With respect to the type of respiratory disorder, consistent associations have been found between exposure to traffic fumes and bronchitis symptoms, while the role of these exposures in the etiology of asthma is still unclear [13].

The SIDRIA project – Studi Italiani sui Disturbi Respiratori nell'Infanzia e l'Ambiente [14-16] – is a large multi-center, population-based study conducted in the framework of the International Study of Asthma and Allergies in Childhood (ISAAC) [17]. The findings from SIDRIA-1, conducted in 1994–1995, showed a positive association between indicators of air pollution from heavy vehicular traffic in the street of residence and a wide range of respiratory disorders in children living in highly urbanized areas [18]. However, it was not possible to assess the independent effects of car and truck traffic in that study as the information was not available. A second phase of SIDRIA was conducted in 2002 to evaluate time trends in the prevalence of respiratory disorders in childhood, according to the ISAAC Phase III protocol [19], to confirm the role of several potential risk factors identified in SIDRIA-1 and to explore some new hypotheses.

Self-reported exposure to road traffic has been recently questioned as a reliable measurement of exposure to air pollutants [20-22]. Heinrich et al [20] compared parental report of traffic intensity at the home address with a combination of air pollution measures and GIS based modelled exposure in two different European countries (Germany and the Netherlands). They found that the degree of agreement between the two methods was relatively low, but the reasons for these discrepancies were not analyzed.

Kuehni et al [21] estimated the association between self-reported exposure to road traffic and respiratory symptoms in preschool children in Leicestershire, UK, and investigated whether the effect could have been caused by reporting bias. The association between traffic exposure and respiratory outcomes was assessed using unconditional logistic regression and conditional regression models (matching by postcode). Matched analysis comparing symptomatic and asymptomatic children living at the same postcode (thus theoretically exposed to similar road traffic) suggested that parents of children with respiratory symptoms reported more road traffic than parents of asymptomatic children.

In this paper, based on the data from SIDRIA-2, we present an analysis of the relation between indicators of road traffic pollution and several chronic respiratory symptoms while evaluating the potential effect of information bias.

## Methods

### Population and study design

The SIDRIA-2 study design has been described elsewhere [15,16,23] and it will be only summarized here. The survey was conducted in 2002, between January and May, in 12 centers (Bari, Colleferro, Emilia-Romagna, Empoli, Florence, Mantova, Milan, Palermo, Rome, Siena, Turin, Trento) of Northern, Center and Southern Italy, different in size, climate, latitude and level of urbanization. Eight of these centers had already participated in SIDRIA-1 [18]. The protocol of the study was approved by Ethics Committee of the Catholic University in Rome.

The sample included 22,442 children (6–7 years old) and 16,336 adolescents (13–14 years old) attending respectively the first two grades of the primary school and the last year of the middle school. The primary sampling units were schools, both public and private, weighted for the number of attending subjects. Each center contributed with at least 1,000 subjects for each age group.

### Data collection

To collect information on the medical history of the children, we used standardized, self administered questionnaires that included also the relevant ISAAC-Phase III questions on asthma, rhinitis and eczema symptoms, and questions on various known or suspected risk factors. For children (6–7 years old), all questionnaires were completed by parents. According to the standard ISAAC protocol [17], the questionnaires for adolescents were filled by adolescents themselves. However, in the SIDRIA study, another questionnaire, including questions on both symptoms and risk factors, was also completed by the adolescents' parents. For reasons of consistency and comparability between age groups, the current analyses are based on the parental questionnaires for both age-groups.

Children with "asthma symptoms" were defined as those reporting in the past 12 months at least one of the following: one or more wheezing episodes, wheeze with exercise, morning chest tightness; or if they reported night dry cough in the last 12 months and had a reporting of lifetime asthma; or those reporting treatment for medically diagnosed asthma or had a hospital admission for asthma in the last 12 months; or if they reported a life time asthma and a positive answer to the question "Is your child still suffering from asthma?".

Children were defined as having "severe asthma" if in the past 12 months at least one of the following were reported: 4 or more wheezing attacks, waking at night with wheezing one or more times a week, an attack severe enough to limit speech to only one or two words at a time between breaths, or a hospital admission for asthma.

Children were defined as having "cough or phlegm" if they reported cough or phlegm for at least 4 days a week (in the absence of a cold) for one or more months a year.

Questions on traffic included a parental subjective evaluation of traffic density in the zone of residence ("absent", "low", "moderate" or "high") and of the daily frequency of passing cars and trucks in the street of residence ("never or seldom", "sometimes", "frequently", "continuously"). The exact wording is reported in the Additional file 1.

### Data analyses

Odds ratio (OR) and 95% confidence intervals (95% CI) were estimated with multiple logistic regression analyses. The basic analyses were for "asthma symptoms" and "cough or phlegm", but we also conducted multinomial logistic regression analyses comparing: i) those with asthma symptoms without cough or phlegm; ii) those with cough or phlegm without asthma symptoms; and iii) those with the combination of the two conditions, with the remaining subjects, negative for both symptoms. For "cough or phlegm", the analyses focused on the possible independent effect of cars and trucks transit in the street of residence. In some of the analyses, in order to obtain sufficient numbers in each category, car transit was recoded into three categories ("absent/sometimes", "frequently" and "continuously") and truck transit was also recoded into three categories ("absent", "sometimes" and "frequently/continuously").

Potential confounding factors included in the multiple logistic regression models were: sex, age, parental asthma or allergy (rhinitis or eczema), parental education (higher educational level between parents as a proxy of socioeconomic status – SES), passive smoke at home (at least one smoker-mother, father or others – in the household), indoor mould/dampness, season, person filling the questionnaire, floor of the apartment, change of residence and study area. Robust estimate of standard errors were used to control for clustering within schools [24].

In order to explore the role of potential effect modifiers, statistical significance of appropriate interaction terms were evaluated through likelihood-ratio tests. Eventually we performed subgroup analyses for different factors (age, gender, latitude, parental education, smoking, parental asthma or allergies, level of urbanization, indoor mould/dampness, floor of the apartment, change of residence), in each case considering the associations of truck traffic exposure (frequent or continuous vs never) and the investigated respiratory symptoms. All analyses were conducted using STATA 9 (Stata Corporation, College Station, Texas).

### Data validation

#### External validation

In the city of Turin data on traffic flows were obtained from the local company of public transportation (5T S.C.R.L), that measures hourly traffic mostly in street segments with medium-high volume of traffic. The address of each subject included in the survey was geocoded and linked to data on traffic using a Geographical Information System (GIS). We excluded 204 subjects (out of 3,453) attending a school in the municipality but resident outside the city of Turin, for which traffic information and geocoding was not available. For each segment of street we calculated average daily vehicle count in work hours (h.07–19) weighted by the number of observations available; this information was linked to the subject using a computer based GIS, excluding subjects living in internal civic numbers. We positively matched 887 subjects out of 3,249 (328 street segments out of 1067). All GIS analyses were performed with the ArcGis software version 9 (ESRI, Redlands, California, USA). We analyzed the frequency distribution for some characteristics of the subjects, including reported traffic, separately for children linked with traffic count and for non-linked subjects. We performed the Kruskal-Wallis test to assess the differences between median traffic counts and calculated Jackknife confidence intervals for the median of the differences [25]. The same analysis was performed for subjects with and without a reporting of current respiratory symptoms, separately for asthmatic symptoms and cough or phlegm (ANOVA) [26].

#### Internal validation

In the 3 metropolitan areas of Turin, Milan and Rome (N = 10,285), we matched the children by census block. The assumption is that within this small area (for example, in Turin a census block covers a mean of 250 subjects – [27]) the true exposure to road traffic in an urban context would be similar; therefore, if the association between traffic exposure and symptoms is true, it should disappear when the comparisons are stratified by census block (matched analysis). For this analysis, only data from census blocks where at least one symptomatic and one asymptomatic subject were living could be used; therefore, a varying number of census block was included in the analysis for each of the respiratory symptoms examined. To further investigate the possibility of a reporting bias, in the subgroup of subjects that could be matched by census block we performed a further analysis having attributed to symptomatic subjects the mean traffic exposure reported by parents of asymptomatic subjects for the same census block; in this way, we theoretically excluded the possibility of a bias due to over-reporting of traffic by parents of symptomatic subjects. In these analyses, reported life time asthma was also included as an endpoint.

## Results

The parental questionnaire was completed for 20,016 6–7 years old (response rate 89.2%) and for 13,616 13–14 years old (response rate 83.3%). Table 1 shows the combined prevalence of asthma symptoms and cough or phlegm. Overall, 13.5% of children (95% CI: 13.2%, 13.9%) were reported to have asthma symptoms, and 6.8% (95% CI: 6.6%, 7.1%) to have cough or phlegm. For 2.9% (95% CI: 2.7%, 3.0%) of the subjects, cough or phlegm and asthma symptoms were concomitant. The frequency of each traffic indicator is presented in the Additional file 2.

**Table 1:** Prevalence of respiratory outcomes investigated in a sample of Italian schoolchildren.

		Asthma symptoms		
**Cough or phlegm**		**YES** n (%)	**NO** n (%)	**TOTAL**
	
	**YES** n (%)	961 (2.9%)	1,337 (4.0%)	2,298 (6.8%)
	
	**NO** n (%)	3,591 (10.7%)	27,743 (82.5%)	31,334 (93.2%)
	
	**TOTAL**	4,552 (13.5%)	29,080 (86.5%)	33,632 (100%)

The prevalences of respiratory symptoms in the various subgroups are shown in Table 2. Respiratory symptoms were more frequent if a parental history of asthma or allergies was reported, among subjects exposed to passive tobacco smoke and in the presence of moulds/dampness in the child's bedroom. The prevalence of respiratory symptoms increased inversely with parental education, and directly with urbanization level.

**Table 2:** Prevalence (%) of current respiratory symptoms by various subgroups (N = 33,632)

	Asthma symptoms % (n)	Cough or phlegm % (n)
**Total**	13.5 (4,552)	6.8 (2,298)

**Age**		

6–7 year olds	13.7 (2,736)	8.4 (1,687)

13–14 year olds	13.3 (1,816)	4.5 (611)

**Gender**		

male	14.6 (2,531)	7.4 (1,275)

female	12.4 (2,012)	6.3 (1,019)

**Parental asthma or allergies**		

yes	17.6 (2,376)	8.9 (1,202)

no	10.8 (2,176)	5.4 (1,096)

**Urbanization level**		

<10,000 inhabitants	11.2 (623)	5.3 (295)

10–100,000 inhabitants	13.2 (1,246)	5.6 (534)

100–500,000 inhabitants	14.8 (973)	7.1 (468)

>500,000 inhabitants	14.2 (1,710)	8.3 (1,001)

**Exposure to passive smoke**		

yes	14.8 (2,509)	7.4 (1,254)

no	12.2 (2,019)	6.2 (1,030)

**Presence of mould/dampness**		

yes	17.4 (631)	8.7 (314)

no	12.9 (3,598)	6.5 (1,807)

*missing*	*14.8 (323)*	*8.1 (177)*

**Parental education**		

primary school	15.1 (207)	7.8 (107)

secondary school	14.2 (1,420)	7.4 (734)

high school	13.3 (2,031)	6.5 (990)

university	12.7 (853)	6.6 (443)

**Floor level of apartment**		

Ground	13.5 (1,273)	6.2 (585)

1^st^–2^nd ^floor	13.9 (1,962)	6.6 (933)

3^rd ^floor or more	12.9 (1,231)	7.7 (738)

**Change of residence**		

Yes	12.6 (2,182)	6.3 (1,099)

No	14.5 (2,174)	7.4 (1,109)

Table 3 shows the associations of traffic indicators with asthma symptoms and with cough or phlegm. Reported high traffic density, continuous car transit and continuous truck transit in the street of residence were weakly associated with asthma symptoms. There were stronger associations of reported high traffic density, continuous car transit and continuous truck transit with cough or phlegm. The associations were generally stronger for truck transit than for car transit. For comparison with the literature, we also present the results of the associations for different asthma symptoms (see Additional file 3). In the same table, we also report the associations separately for light and severe asthma symptoms, and for symptoms of cough or phlegm of different duration (1–2 months a year and 3 or more months a year). Light and severe asthmatic symptoms, as well as symptoms of cough or phlegm of different persistence, did not show a clear pattern with traffic indicators; only for truck traffic the data suggest a trend for stronger associations in subjects with more severe symptoms.

**Table 3:** Associations between traffic indicators and asthma symptoms and cough or phlegm

	Asthma symptoms				Cough or phlegm			
	**n cases**	**(%)**	OR*	**95% CI**	**n cases**	**(%)**	OR*	**95% CI**

**Traffic density**								

Absent	564	12.5	1.00		270	6.0	1.00	

Low	1,228	12.6	1.04	0.93–1.16	558	5.7	0.94	0.80–1.10

Moderate	1,375	13.8	1.13	1.01–1.26	689	6.9	1.04	0.89–1.22

High	795	14.6	1.17	1.03–1.33	475	8.7	1.24	1.04–1.49

**Frequency cars transit**								

Never	309	12.7	1.00		142	5.9	1.00	

Sometimes	1,147	12.1	0.95	0.83–1.10	517	5.5	0.90	0.74–1.10

Frequently	1,399	13.6	1.06	0.93–1.22	679	6.6	1.02	0.84–1.26

Continuously	1,108	14.9	1.16	1.00–1.33	656	8.8	1.32	1.08–1.63

**Frequency trucks transit**								

Never	1,412	12.3	1.00		652	5.7	1.00	

Sometimes	1,644	13.6	1.10	1.02–1.19	812	6.7	1.14	1.02–1.26

Frequently	682	14.5	1.16	1.05–1.29	399	8.5	1.41	1.23–1.61

Continuously	211	15.7	1.27	1.08–1.50	132	9.8	1.67	1.36–2.06

* All ORs were adjusted for study centre, age, sex, parental asthma or allergies, parental education, passive smoke at home, indoor moulds, season, person filling the questionnaire, floor level of the apartment and change of residence.

The relationship between these traffic indicators and the respiratory outcomes was explored in more detail evaluating the associations with asthma symptoms without cough or phlegm, cough or phlegm without asthma and the combination of the two conditions (Table 4). Asthma symptoms, when alone, were only weakly associated with high traffic density, continuous car and continuous truck transit. Traffic exposure indicators were more strongly associated with cough or phlegm, and particularly when accompanied by asthma symptoms. For this last condition (asthma with cough or phlegm) there were strong and significant associations for all the indicators.

**Table 4:** Associations between traffic indicators and asthma symptoms with or without cough or phlegm.

	Asthma symptoms WITHOUT cough or phlegm				Cough or phlegm WITHOUT asthma symptoms				Asthma symptoms WITH cough or phlegm			
	**n cases**	**(%)**	OR*	**95% CI**	**n cases**	**(%)**	OR*	**95% CI**	**n cases**	**(%)**	OR*	**95% CI**

**Traffic density**												

Absent	465	10.3	1.00		171	3.8	1.00		99	2.2	1.00	

Low	996	10.2	1.00	0.89–1.13	326	3.4	0.83	0.69–1.00	232	2.4	1.12	0.88–1.42

Moderate	1,083	10.9	1.08	0.95–1.22	397	4.0	0.92	0.75–1.11	292	2.9	1.27	1.02–1.57

High	601	11.0	1.13	0.99–1.28	281	5.2	1.14	0.92–1.41	194	3.6	1.52	1.17–1.96

**Frequency cars transit**												

Never	254	10.5	1.00		87	3.6	1.00		55	2.3	1.00	

Sometimes	938	9.9	0.93	0.80–1.07	308	3.3	0.91	0.72–1.15	209	2.2	0.97	0.71–1.34

Frequently	1,129	11.0	1.03	0.89–1.19	409	4.0	1.07	0.83–1.36	270	2.6	1.10	0.80–1.52

Continuously	823	11.0	1.07	0.92–1.25	371	5.0	1.30	1.02–1.67	285	3.8	1.53	1.11–2.10

**Frequency trucks transit**												

Never	1,158	10.1	1.00		398	3.5	1.00		254	2.2	1.00	

Sometimes	1,299	10.8	1.07	0.98–1.17	467	3.9	1.10	0.95–1.26	345	2.9	1.27	1.09–1.48

Frequently	525	11.1	1.13	1.01–1.26	242	5.1	1.44	1.23–1.69	157	3.3	1.51	1.21–1.87

Continuously	150	11.2	1.21	1.01–1.46	71	5.3	1.47	1.13–1.93	61	4.5	2.11	1.58–2.81

* All ORs were adjusted for study centre, age, sex, parental asthma or allergies, parental education, passive smoke at home, indoor moulds, season, person filling the questionnaire, floor level of the apartment and change of residence.

The analyses of the independent and joint effects of truck traffic and car traffic on the risk of cough or phlegm are shown in Table 5. In some categories the numbers are relatively small and the effect estimates are therefore unstable. The table confirms an independent effect of truck transit on cough or phlegm (OR = 1.50; 95% CI: 0.93, 2.43), and also shows a similar association for car transit (OR = 1.30; 95% CI: 0.94, 1.81).

**Table 5:** Associations between combined exposure to truck and car transit and cough or phlegm.

		Trucks transit			
			**Never**	**Sometimes**	**Freq/continuously**

**Cars transit**	**Never/sometimes**	*n cases (%)*	*426 (5.3)*	*211 (5.9)*	*19 (8.2)*
		
		OR* (95% CI)	1.00	1.12 (0.95–1.32)	1.50 (0.93–2.43)
	
	**Frequently**	*n cases (%)*	*168 (6.3)*	*370 (6.4)*	*140 (7.7)*
		
		OR* (95% CI)	1.09 (0.90–1.33)	1.12 (0.96–1.30)	1.42 (1.15–1.75)
	
	**Continuously**	*n cases (%)*	*56 (7.8)*	*228 (8.4)*	*372 (9.3)*
		
		OR* (95% CI)	1.30 (0.94–1.81)	1.42 (1.19–1.70)	1.60 (1.36–1.87)

*All ORs were adjusted for presence of current asthma symptoms, study centre, age, sex, parental asthma or allergies, parental education, passive smoke at home, indoor moulds, season, person filling the questionnaire, floor level of the apartment and change of residence.

**Figure 1:**
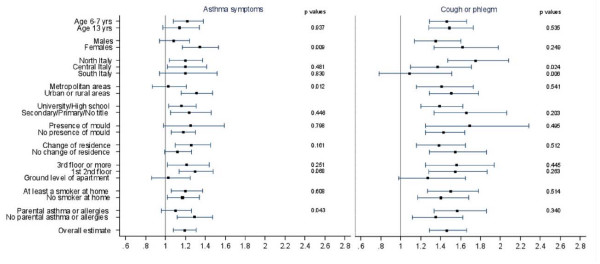
**Associations between exposure to truck transit and respiratory symptoms, by several characteristics**. The figure reports the associations (OR and 95% CI) between exposure to truck transit (frequent/continuous *vs *never) and asthma symptoms and cough or phlegm, by different factors (age, gender, latitude, level of urbanization, parental education, indoor mould/dampness, change of residence, floor of the apartment, passive smoke at home, parental asthma or allergies). All ORs were adjusted for potential confounder, excluding the stratification factor. Statistical significance (p values) of the interaction terms are reported. SIDRIA-2.

The results of the analyses performed to explore the possible effect modification of the association with frequent or continuous truck transit are reported in Figure 1. Higher risks were observed for females for asthma symptoms (OR = 1.34; 95% CI: 1.17, 1.53) and cough or phlegm (OR = 1.62; 95% CI: 1.33, 1.98) than for males (OR = 1.08; 95% CI: 0.94,1.24; and OR = 1.35; 95% CI: 1.14, 1.60 respectively). The pattern was present for both age groups. Cough or phlegm were more associated with truck transit in the Northern areas of the Country (OR = 1.75; 95% CI: 1.47, 2.08) than in the Central and in the Southern areas (OR = 1.37; 95% CI: 1.10, 1.71 and OR = 1.09; 95% CI: 0.78, 1.51). Asthma symptoms were more associated with truck transit in the urban or rural areas (OR = 1.31; 95% CI: 1.16, 1.47) than in the metropolitan areas (i.e. municipalities with more than 500.000 inhabitants) (OR = 1.03; 95% CI: 0.87, 1.21). Asthma symptoms were more associated with truck transit when parental asthma or allergies were not reported. Comparable results were observed for the indicator of continuous car traffic (see Additional file 4).

### Results of the external validation

The subgroup of subjects with measured traffic data in Turin had a significant higher exposure to all the reported traffic indicators (p < 0.0001, see Additional file 5). This was an expected result, given that traffic data are available mostly for streets with high traffic intensity. In this subgroup, a lower prevalence of asthmatic symptoms was observed; no statistically significant associations were observed between traffic count and respiratory symptoms, although for cough or phlegm the adjusted OR for the highest quintile of traffic exposure (more than 22,732 vehicles/day) was 1.48 (95%CI 0.80–2.73) compared to the lowest quintile (less than 5,608 vehicles/day).

**Figure 2:**
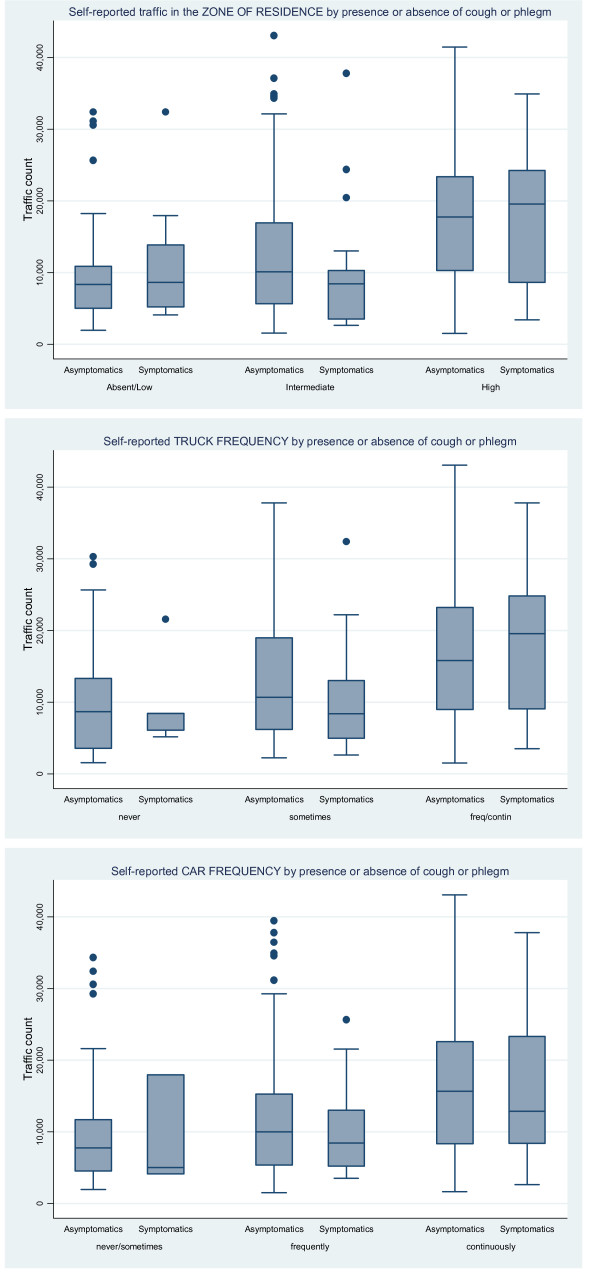
**Box Plot of the daily number of vehicles by categories of traffic indicators**. The figure reports the daily number of vehicles by traffic in the zone of residence (upper), truck transit in the street of residence (intermediate) and car transit in the street of residence (lower), by presence or absence of symptoms of cough or phlegm. SIDRIA-2 Turin.

All traffic indicators from questionnaire were associated with the daily median number of vehicles passing in work hours: for truck frequency "never or seldom" = 8,549 (95% CI: 6,500, 9,073), "sometimes" = 10,626 (95% CI: 9,580, 11,525), "frequently" = 14,359 (95% CI: 12,848, 15,834), "always or nearly always" = 19,120 (95% CI: 16,817, 22,618); the same was observed for the three categories of traffic density in the zone: "absent\low" = 8,459 (95% CI: 5,332, 9,073), "intermediate" = 10,109 (95% CI: 8,857, 10,692), "high" = 17,745 (95% CI: 15,814, 18,722) and for car frequency: "never\sometime" = 7,378 (95% CI: 5,042, 9,566), "frequently" = 9,840 (95% CI: 8,914, 10,692), "always or nearly always" = 15,277 (95% CI: 13,664, 16,873). The Kruskal-Wallis test for median traffic count by categories of each traffic question was always highly significant (p < 0.0001). The same analysis was performed separately for subjects with and without a reporting of current respiratory symptoms. Figure 2 reports the results of this analysis for subjects with and without symptoms of cough or phlegm; at the ANOVA test there were no differences between the two groups of symptomatic and asymptomatic subjects in the daily median number of vehicles passing in the road of residence. Similar results were obtained when we analysed separately the subjects with and without a reporting of asthmatic symptoms (data not shown).

### Results of the internal validation

In the overall sample recruited in the cities of Turin, Milan and Rome, we obtained valid addresses for 88.8% out of 10,285 subjects. A total of 9,034 children were included in the analysis (whole sample) whereas 2,446 subjects could be included (498 census blocks out of 4,210) in the matched analysis by census block for cough or phlegm (see Additional files 6 and 7). The subgroup considered for the matched analysis differed from the whole sample for parental education (lower), age (younger) and exposure to traffic in the zone of residence (lower). Similar differences were present in the subgroup of the matched analysis for asthmatic symptoms (3,086 subjects, data not shown).

**Figure 3:**
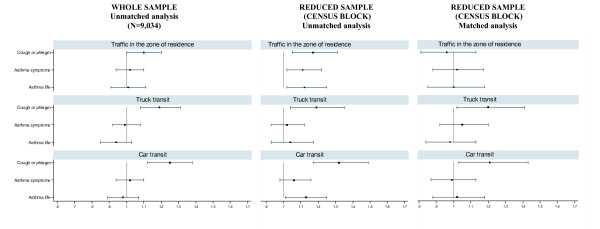
**Associations (unmatched and matched logistic regression) between self-reported traffic indicators and respiratory symptoms**. Results (OR and 95% CI) are reported for the whole sample (first column) and the subgroup of subjects that could be matched by census block (unmatched analysis: second column; matched analysis: third column). SIDRIA-2 Rome, Turin and Milan.

In the whole sample, significant associations were observed for symptoms of cough or phlegm for all the three indicators of exposure, while non significant positive associations were present for current asthma symptoms or lifetime asthma (Figure 3-first column). Figure 3 (second column) shows the associations (unmatched analysis) between traffic indicators and respiratory symptoms in the subgroup of subjects that could be matched by census blocks: a similar pattern compared with those of the whole sample (first column) was found for symptoms of cough or phlegm, while higher risks were present for asthma symptoms and for lifetime asthma. The results of the matched regression analysis (Figure 3-third column) differed according to the traffic indicator examined. When traffic density in the zone of residence was considered, no association was still present, for all the endpoints. When the two indicators of the traffic in the street of residence were considered, the ORs remained positive and statistically significant for cough or phlegm.

**Figure 4:**
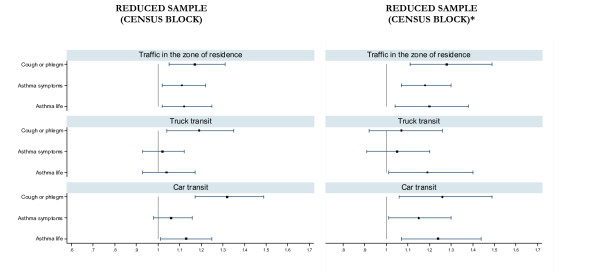
**Associations (unmatched logistic regression, OR and 95% CI) between self-reported traffic indicators and respiratory symptoms**. Results are reported for the subgroup of subjects that could be matched by census block using original data on reported traffic (first column) and assigning to cases in each census block the average traffic reported by controls (second column*). SIDRIA-2 Rome, Turin and Milan.

The results of the logistic regression analysis in which, for each census block, we attributed to symptomatic subjects the mean traffic exposure reported by parents of asymptomatic subjects are reported in Figure 4 (second column). For all the outcomes and all the traffic indicators (except for the comparison between cough or phlegm and truck traffic) the associations remained stable and the point estimates were often higher than those observed using original data (Figure 4-first column).

We conducted a final sensitivity analysis where we matched subjects by street of residence. This was done to overcome the fact that exposure levels within census blocks could be not homogenous. However, this procedure of matching generated a selected sample with a higher exposure to reported traffic compared to the whole sample. Furthermore, in the unmatched analysis of the subgroup there was no positive significant association between the traffic indicators and respiratory symptoms (all the Odds Ratios were close to 1 or even negative) thus precluding the possibility to learn from the matched analysis (data not shown).

## Discussion

This study confirms our previously-reported associations between frequency and density of traffic in the street of residence and current respiratory symptoms in children [18]. For all indicators of traffic density and frequency, an increase in symptoms by level of exposure was observed. The strongest associations were for cough or phlegm and truck traffic, although an independent effect of continuous car traffic was found. Traffic fume exposure was weakly associated with asthma symptoms, but the association was stronger if asthma was accompanied by cough or phlegm.

The results of the external validation did not support the existence of a reporting bias for the observed associations, for all the self-reported traffic indicators examined. The internal validation indicates that a response bias is not responsible of the associations between traffic density in the zone of residence and respiratory symptoms, while an over-reporting of truck traffic by parents of symptomatic subjects cannot be excluded.

### Strengths and limitations

The main strength of the present study is that it was based on a large random general population sample with a high response rate (86.7%). The information on symptoms of asthma and cough or phlegm included items derived from standardized validated questionnaires, that have been used in previous epidemiological studies in this population [28] and internationally [17].

The main weakness of our study is that it relies for both symptoms and exposure on self-reported information. In this study, the validity of information on traffic exposure was deeply evaluated in order to examine the existence of a possible reporting bias due to an over-reporting of traffic intensity by parents of symptomatic children, suggested by some authors [21].

Kuehni et al [21] reported that parents of children with more severe respiratory problems (i.e. asthma diagnosis and bronchodilator use) are particularly prone to overestimate traffic exposure, given that they have "received the broadest media coverage with regard to air pollution". However, in the total sample included in the SIDRIA 2 study, we consistently found the strongest associations with traffic indicators for symptoms of cough or phlegm rather than for asthma symptoms; furthermore, severe and light asthmatic symptoms did not show a different association with traffic indicators. The validation analyses consistently did not show evidence of an over-reporting of traffic by parents of children for which asthma was reported.

The study of Piro et al [22] suggested that subjects with a chronic diseases tend to report more air pollution problems in area of living. In our study (Figure 1), parental asthma or allergy did not act as modifiers of the association between cough or phlegm and truck traffic; furthermore, for asthma symptoms, the association was stronger among parents that did not report asthma or allergies. Other studies have found that the association between exposure to traffic and asthma are higher among children with no reported family history [29,30] or maternal history of asthma [31]. These findings are reassuring with regards to the possibility of a reporting bias due to an over-reporting of traffic information among parents suffering from asthma or allergic disorders.

In the subgroup of children for which measurements of traffic in the segment of street of residence were available, we observed that the daily median count of vehicles was well predicted by the categories of traffic indicators reported by parents, without systematic differences between symptomatic and asymptomatic children. A similar finding was observed in the same city in the first phase of the SIDRIA study [18]. These results do not support the existence of a reporting bias for the observed associations, for each of the traffic indicators and each of the respiratory symptoms examined. The major limitations of this external validation are that it was possible in a single centre and it was based on a small and selected group of highly exposed subjects that could be linked with an objective measure.

Applying a method comparable to that used by Kuehni et al [21] where 7-digit postal code was used as a variable of matching, in the analyses in which subjects were matched by census block we observed that the associations between reported traffic (trucks or cars) in the street of residence and symptoms of cough or phlegm did not disappear (Figure 3). On the contrary, the associations disappeared when the reported traffic in the zone or residence (that is, an area based indicator) was examined, for each of the respiratory symptoms. These results could imply that parents of subjects with symptoms of cough or phlegm could over-report the traffic in the street of residence. However, another plausible explanation for these findings is the presence of a residual variability in the type of roads within the same census block. In the subgroup of subjects considered for the matched analysis we attributed to symptomatic subjects the mean traffic exposure reported by parents of asymptomatic subjects (in this way, we excluded the possibility of a bias due to over-reporting of traffic by parents of symptomatic subjects). The results of this analysis (Figure 4) do not support the presence of response bias for car traffic and traffic in the zone of residence, although do not allow to definitively exclude an over-reporting of truck traffic by parents of subjects with cough or phlegm (but not with current or lifetime asthma).

One of the main limitation of the matching procedure (according to which at least one symptomatic and one asymptomatic subject have to be present in each census block) is the small number of subjects included in the analyses. Furthermore, the subjects included in these subgroups can be selected, i.e. no more representative of the whole population. Despite the limitation of the method used for internal validation, the overall results do not seem to support the existence of a relevant response bias. The results suggest that, if a bias is present, this should be due to an over-reporting of truck traffic (but not of other traffic indicators) by parents of subjects with symptoms of cough or phlegm (but not of asthma).

The possibility of important residual confounding in our results is low as we adjusted for several known or suspected risk factors for respiratory symptoms; however, we lacked some outdoor and indoor information, like ventilation characteristics of the house. The times when windows were typically opened differed between Northern and Central Italy [32], though the magnitude of the effect was small on indoor air pollutants concentrations. Our questionnaire was focused on traffic pollution in the street and zone of residence, but did not collect information about other exposures such as those close to the schools. The absence of these details could have led to a small random misclassification of the exposures in the participants, while it seems unlikely as an explanation of the observed positive associations.

For reasons of consistency and comparability between age groups, we based the analyses on the parental questionnaires for both age-groups. However, in a sensitivity analysis we examined the associations between traffic indicators and respiratory symptoms including the answers on symptoms given by adolescents (see Additional file 8). The results for the outcome of cough or phlegm were substantially unchanged.

### Interpretation of the results

Few published studies have analyzed the effects of traffic in relationship to combinations of respiratory outcome such as asthma symptoms and persistent cough or phlegm. A positive association of air pollution with bronchitis symptoms, but not with asthma, has been reported in some studies [3,6]. Other studies found both associations; e.g. Kim et al. [33] found a significant increase in the risk of bronchitic symptoms and asthma in relation to higher level of traffic related to pollution in a US area with relatively clean air [33]. However, the findings of different studies should be compared with caution, because the definition of asthma adopted may be different and it can often include symptoms that may be related to bronchitis rather than to asthma [34]. In fact we have found an increased risk of asthma symptoms in children exposed to car and truck traffic fumes, essentially restricted to those who also reported cough or phlegm. There are at least two possible explanations for these findings. Firstly, it is possible that these children only had chronic bronchitis and did not have asthma symptoms, i.e. that they were "misdiagnosed" by our asthma symptom questionnaire [35]. The second, and perhaps more likely explanation, is that these children experienced an increased risk for a form of asthma that also involved persistent cough and phlegm.

Several previous questionnaire-based studies and surveys that used traffic counts as exposure metrics, have suggested that heavy traffic powered by diesel engines is more harmful for respiratory health than light traffic powered by gasoline engines [13,34]. When we analyzed the effects of car and truck traffic separately on cough or phlegm, we found a stronger association with heavy traffic exposure (Table 4), consistent with the findings from other studies [4,7,9].

We found significant associations of car traffic with cough or phlegm, although these were generally weaker than the associations for truck traffic (Tables 4 and 5). These findings could indicate that car traffic may also represent a source of pollutants increasing the risk of chronic cough or phlegm. However, these findings could also be related to the very high, and still increasing, number of cars powered by diesel engines at present circulating in Italy [36], and does not necessarily indicate that other types of car fume exposure also increase the risk of respiratory symptoms. Children exposed to both, continuous car traffic and frequent or continuous truck transit, showed the highest risk of cough or phlegm (Table 5). This is consistent with the hypothesis that truck and car traffic involve similar exposures (e.g. diesel fumes).

With respect to exposure to traffic and related respiratory outcomes, we found stronger associations in females, for both age groups. An increased susceptibility of girls to air pollution has been reported by several investigators [4,9,10,33] and was partially observed also in SIDRIA-1 [18]. A higher pollutant-to-lung volume ratio that results from larger airways relative to lung size in girls than in boys and enhanced cholinergic irritability could be considered as possible explanations [37,38]. A gradient in the geographical distribution of the ORs for truck exposure and cough or phlegm is present in our data. This trend is consistent with the hypothesis that geo-climatic factors characterizing the Northern regions do favor accumulation of atmospheric pollutants. In contrast with the findings of the previous phase of SIDRIA, for symptoms of cough or phlegm we did not observe stronger associations among subjects living in metropolitan areas (more than 500,000 inhabitants) compared to those living in less urbanized areas; on the contrary, stronger associations were observed for asthma symptoms among urban-rural areas (less than 500,000 inhabitants). A possible interpretation may be the presence of higher contrasts in pollution levels in urban-rural areas than in metropolitan areas.

## Conclusion

Although the specific set of airborne toxicants that facilitate and promote respiratory effects are still not known, our findings add further evidence to support a causal effect of exposure to traffic road pollution on respiratory illnesses in childhood. Despite the limitations of the methods used for data validation, it is unlikely that a reporting bias would be responsible for the association we found with traffic exposure in the area of residence. However, since population characteristics are specific, the results of validation of self-reported traffic exposure can not be generalized.

## List of abbreviations

OR: Odds Ratio; CI: Confidence Intervals; ISAAC: International Study of Asthma and Allergies in Childhood; SIDRIA: Studi Italiani sui Disturbi Respiratori nell'Infanzia e l'Ambiente; GIS: Geographical Information System; SES: Socio-economic status; ANOVA: Analysis of Variance.

## Competing interests

The authors declare that they have no competing interests.

## Authors' contributions

EM assisted in the planning of the study, data collection, data management and analyses and drafted the manuscript. GB was involved in statistical analyses, interpretation of the results, and helped drafting the manuscript. CG was national coordinator of the study, conceived the study and participated in its design and conduction and in the manuscript preparation. NP contributed to data analyses, interpretation of the results, and critical review of the manuscript. FF, LB, ECh, GG, PS, VDO, LA, CG and GC were local coordinator and were involved in the conception and design of the study, acquisition of local data, interpretation of the results, and critical review of the manuscript. RC was involved in statistical analyses and performed the GIS analyses. AB and GV participated in the study design, statistical analyses, and revision of the manuscript. MB, RP and LA, as respiratory specialists, were involved in medical data collection, interpretation of the results and revision of the paper. EC, as air quality specialist, contributed to exposure assessment and critical review of the manuscript. All of the authors have read and approved the final version of the manuscript.

## Supplementary Material

Additional file 1 **Wording of the traffic questions**. The table reports the exact wording of the questions on exposure to traffic. Click here for file

Additional file 2 **Traffic exposure in the whole SIDRIA 2 sample**. Frequency (numbers and percentages) of reported exposure by each traffic indicator. Click here for file

Additional file 3 **Associations between traffic indicators and selected respiratory symptoms**. The table shows the results of the associations (odds ratios) for: 1. different asthma symptoms; 2. light and severe asthma symptoms; 3. symptoms of cough or phlegm of different duration (1–2 months per year and 3 or more months per year). Click here for file

Additional file 4 **Associations between exposure to car transit and respiratory symptoms, by several characteristics**. The figure reports the associations (OR and 95% CI) between exposure to car transit (continuous vs never/sometimes) and asthma symptoms and cough or phlegm by different factors (age, gender, latitude, level of urbanization, parental education, indoor mould/dampness, change of residence, floor of the apartment, passive smoke at home, parental asthma or allergies). All ORs were adjusted for potential confounder, excluding the stratification factor. Statistical significance (p values) of the interaction terms are reported. Click here for file

Additional file 5 **Characteristics of subjects geocoded in the city of Turin**. Distribution of frequencies in subgroups linked and not linked with traffic measurements. Click here for file

Additional file 6 **Characteristics of subjects (cities of Turin, Milan and Rome) included in the internal validation analysis**. Description of the whole sample (N = 9,034) and of the subgroup of subjects that could be matched by census block (N = 2,446) for "cough or phlegm". Click here for file

Additional file 7 **Characteristics of subjects (cities of Turin, Milan and Rome) included in the internal validation analysis**. Reported exposure to traffic in the whole sample (first column). For the symptom of "cough or phlegm", exposure to traffic in the subgroups of subjects that could be matched by census block (in which at least one symptomatic and one asymptomatic subject were living), and in the subgroups of subjects living in census blocks where only asymptomatic subjects and only symptomatic subjects were present. Click here for file

Additional file 8 **Associations between traffic indicators in the overall sample (n = 33,632), by source of information. SIDRIA 2**. Table A: for both age groups (6–7 years and 13–14 years), information on exposure and symptoms are those reported by parents. Table B: for the younger age group (6–7 years), information on exposure and symptoms are those reported by parents, while for the older age group (13–14 years), the exposure is reported by parents and symptoms are reported by adolescents. Click here for file
