# Supplementary material for: Respiratory symptoms in children living near busy roads and their relationship to vehicular traffic: results of an Italian multicenter study (SIDRIA 2)
Source: Environ Health. 2009 Jun 18;8:27. doi: 10.1186/1476-069X-8-27 (PMC2708149; doi:10.1186/1476-069X-8-27)
Supplement: Additional file 1 — Wording of the traffic questions. The table reports the exact wording of the questions on exposure to traffic. [file 1476-069X-8-27-S1.doc]

**Wording of the traffic questions**

The table reports the exact wording of the questions on exposure to traffic.

1) Your house is placed in a zone

- without traffic (absent)
- with low traffic (low)
- with moderate traffic (moderate)
- with high traffic (high)

2) In the street where you live, is there truck traffic on working days?

- never or seldom
- sometimes
- frequently, for most of the day
- continuously, always or nearly always during the day

3) In the street where you live, is there car traffic?

- never or seldom
- sometimes
- frequently, for most part of the day
- continuously, always or nearly always during the day
