# Supplementary material for: Respiratory symptoms in children living near busy roads and their relationship to vehicular traffic: results of an Italian multicenter study (SIDRIA 2)
Source: Environ Health. 2009 Jun 18;8:27. doi: 10.1186/1476-069X-8-27 (PMC2708149; doi:10.1186/1476-069X-8-27)
Supplement: Additional file 2 — Traffic exposure in the whole SIDRIA 2 sample. Frequency (numbers and percentages) of reported exposure by each traffic indicator. [file 1476-069X-8-27-S2.doc]

**Traffic exposure in the whole SIDRIA 2 sample**

Frequency (numbers and percentages) of reported exposure by each traffic indicator.

|  | **N** | **%** |
| --- | --- | --- |
| **Traffic density** | |  |
| Absent | 5,127 | 15.2 |
| Low | 10,861 | 32.3 |
| Moderate | 11,032 | 32.8 |
| High | 6,039 | 18.0 |
| *missing* | *573* | *1.7* |
| **Frequency cars transit** | | |
| Never | 2,737 | 8.1 |
| Sometimes | 10,561 | 31.4 |
| Frequently | 11,363 | 33.8 |
| Continuously | 8,351 | 24.8 |
| *missing* | *620* | *1.8* |
| **Frequency trucks transit** | | |
| Never | 12,669 | 37.7 |
| Sometimes | 13,478 | 40.1 |
| Frequently | 5,268 | 15.7 |
| Continuously | 1,555 | 4.6 |
| *missing* | *662* | *2.0* |
| **Whole sample** | **33,632** |  |
