# Supplementary material for: Respiratory symptoms in children living near busy roads and their relationship to vehicular traffic: results of an Italian multicenter study (SIDRIA 2)
Source: Environ Health. 2009 Jun 18;8:27. doi: 10.1186/1476-069X-8-27 (PMC2708149; doi:10.1186/1476-069X-8-27)
Supplement: Additional file 3 — Associations between traffic indicators and selected respiratory symptoms. The table shows the results of the associations (odds ratios) for: 1. different asthma symptoms; 2. light and severe asthma symptoms; 3. symptoms of cough or phlegm of different duration (1–2 months per year and 3 or more months per year). [file 1476-069X-8-27-S3.doc]

**Associations between traffic indicators and selected respiratory symptoms.**

The table shows the results of the associations (odds ratios) for: 1. different asthma symptoms; 2. light and severe asthma symptoms; 3. symptoms of cough or phlegm of different duration (1-2 months per year and 3 or more months per year).

*All ORs were adjusted for study centre, age, sex, parental asthma or allergies, parental education, passive smoke at home, indoor moulds, season, person filling the questionnaire, floor level of the apartment and change of residence.
