# Supplementary material for: Respiratory symptoms in children living near busy roads and their relationship to vehicular traffic: results of an Italian multicenter study (SIDRIA 2)
Source: Environ Health. 2009 Jun 18;8:27. doi: 10.1186/1476-069X-8-27 (PMC2708149; doi:10.1186/1476-069X-8-27)
Supplement: Additional file 4 — Associations between exposure to car transit and respiratory symptoms, by several characteristics. The figure reports the associations (OR and 95% CI) between exposure to car transit (continuous vs never/sometimes) and asthma symptoms and cough or phlegm by different factors (age, gender, latitude, level of urbanization, parental education, indoor mould/dampness, change of residence, floor of the apartment, passive smoke at home, parental asthma or allergies). All ORs were adjusted for potential confounder, excluding the stratification factor. Statistical significance (p values) of the interaction terms are reported. [file 1476-069X-8-27-S4.doc]

**Associations between exposure to car transit and respiratory symptoms, by several characteristics.**

The figure reports the associations (OR and 95% CI) between exposure to car transit (continuous vs never/sometimes) and asthma symptoms and cough or phlegm by different factors (age, gender, latitude, level of urbanization, parental education, indoor mould/dampness, change of residence, floor of the apartment, passive smoke at home, parental asthma or allergies). All ORs were adjusted for potential confounder, excluding the stratification factor. Statistical significance (p values) of the interaction terms are reported.
