# Supplementary material for: Respiratory symptoms in children living near busy roads and their relationship to vehicular traffic: results of an Italian multicenter study (SIDRIA 2)
Source: Environ Health. 2009 Jun 18;8:27. doi: 10.1186/1476-069X-8-27 (PMC2708149; doi:10.1186/1476-069X-8-27)
Supplement: Additional file 5 — Characteristics of subjects geocoded in the city of Turin. Distribution of frequencies in subgroups linked and not linked with traffic measurements. [file 1476-069X-8-27-S5.doc]

**Characteristics of subjects geocoded in the city of Turin.**

Distribution of frequencies in subgroups linked and not linked with traffic measurements.

|  |  | **Not linked group (N=2,362)** | | **Linked group (N=887)** | |
| --- | --- | --- | --- | --- | --- |
|  |  | **N** | **%** | **N** | **%** |
| Sex |  |  |  |  |  |
|  | Male | 1,253 | 53.0 | 468 | 52.8 |
|  | Female | 1,109 | 47.0 | 419 | 47.2 |
| Age |  |  |  |  |  |
|  | 6-7 years | 1,638 | 69.3 | 586 | 66.1 |
|  | 13-14 years | 724 | 30.7 | 301 | 33.9 |
| Questionnaire filled in by | |  |  |  |  |
|  | Mother | 1,246 | 52.8 | 501 | 56.5 |
|  | Other | 1,065 | 45.1 | 365 | 41.1 |
| Parental education | |  |  |  |  |
|  | University/High school | 1,477 | 62.5 | 553 | 62.3 |
|  | Secondary/Primary/No title | 865 | 36.6 | 324 | 36.5 |
| Asthma symptoms | |  |  |  |  |
|  | Yes | 345 | 14.6 | 96 | 10.8 |
|  | No | 2,017 | 85.4 | 791 | 89.2 |
| Cough or phlegm | |  |  |  |  |
|  | Yes | 214 | 9.1 | 78 | 8.8 |
|  | No | 2,148 | 90.9 | 809 | 91.2 |
| In the zone of residence | |  |  |  |  |
|  | Traffic density: |  |  |  |  |
|  | absent/low | 640 | 27.1 | 77 | 8.7 |
|  | moderate | 1,103 | 46.7 | 302 | 34.0 |
|  | high | 561 | 23.8 | 505 | 56.9 |
|  | *Missing* | *58* | 2.5 | *3* | 0.3 |
| In the street of residence | |  |  |  |  |
|  | Daily lorry transit: |  |  |  |  |
|  | - never | 648 | 27.4 | 68 | 7.7 |
|  | - sometime | 1,170 | 49.5 | 324 | 36.5 |
|  | - frequently | 397 | 16.8 | 351 | 39.6 |
|  | - continuously | 88 | 3.7 | 140 | 15.8 |
|  | *- Missing* | *59* | 2.5 | *4* | 0.5 |
|  | Daily cars transit: |  |  |  |  |
|  | - never/sometimes | 563 | 23.8 | 51 | 5.7 |
|  | - frequently | 1,028 | 43.5 | 205 | 23.1 |
|  | - continuously | 714 | 30.2 | 629 | 70.9 |
|  | *- Missing* | *57* | 2.4 | *2* | 0.2 |
|  | **Total** | **2,362** | **100.0** | **887** | **100.0** |
