# Supplementary material for: Respiratory symptoms in children living near busy roads and their relationship to vehicular traffic: results of an Italian multicenter study (SIDRIA 2)
Source: Environ Health. 2009 Jun 18;8:27. doi: 10.1186/1476-069X-8-27 (PMC2708149; doi:10.1186/1476-069X-8-27)
Supplement: Additional file 6 — Characteristics of subjects (cities of Turin, Milan and Rome) included in the internal validation analysis. Description of the whole sample (N = 9,034) and of the subgroup of subjects that could be matched by census block (N = 2,446) for "cough or phlegm". [file 1476-069X-8-27-S6.doc]

**Characteristics of subjects (cities of Turin, Milan and Rome) included in the internal validation analysis.**

Description of the whole sample (N=9,034) and of the subgroup of subjects that could be matched by census block (N=2,446) for “cough or phlegm”.

|  |  | **Whole sample (N=9034)** | | **Subgroup that could be matched by census block *** | |
| --- | --- | --- | --- | --- | --- |
|  |  | **N** | **%** | **N** | **%** |
| Sex |  |  |  |  |  |
|  | - Male | 4,647 | 51.4 | 1,313 | 53.7 |
|  | - Female | 4,387 | 48.6 | 1,133 | 46.3 |
| Age |  |  |  |  |  |
|  | -6-7 years | 5,944 | 65.8 | 1,832 | 74.9 |
|  | - 13 year | 3,090 | 34.2 | 614 | 25.1 |
| Questionnaire filled in by | |  |  |  |  |
|  | - Mother | 5,167 | 57.2 | 1,375 | 56.2 |
|  | - Other | 3,691 | 40.9 | 1,020 | 41.7 |
| Parental education | |  |  |  |  |
|  | University | 2,414 | 26.7 | 466 | 19.1 |
|  | High school | 4,075 | 45.1 | 1,141 | 46.6 |
|  | Secondary | 2,228 | 24.7 | 736 | 30.1 |
|  | Primary/no title | 237 | 2.6 | 77 | 3.1 |
| At least one smoker at home | |  |  |  |  |
|  | - Yes | 4,748 | 52.6 | 1,308 | 53.5 |
|  | - No | 4,238 | 46.9 | 1,123 | 45.9 |
| Mould | |  |  |  |  |
|  | - Yes | 628 | 7.0 | 201 | 8.2 |
|  | - No | 7,863 | 87.0 | 2,074 | 84.8 |
| Parental asthma or allergy | |  |  |  |  |
|  | - Yes | 3,962 | 43.9 | 1,074 | 43.9 |
|  | - No | 5,072 | 56.1 | 1,372 | 56.1 |
| Cough or phlegm | |  |  |  |  |
|  | - Yes | 794 | 8.8 | 606 | 24.8 |
|  | - No | 8,240 | 91.2 | 1,840 | 75.2 |
| Asthma symptoms | |  |  |  |  |
|  | - Yes | 1,270 | 14.1 | 435 | 17.8 |
|  | - No | 7,764 | 85.9 | 2,011 | 82.2 |
|  |  |  |  |  |  |
|  | **Total** | **9,034** | **100.0** | **2,446** | **100.0** |

* Proportions are calculated in the subgroups used for matched analyses on cough or phlegm (498 census blocks out of 4,210).
