# Supplementary material for: Respiratory symptoms in children living near busy roads and their relationship to vehicular traffic: results of an Italian multicenter study (SIDRIA 2)
Source: Environ Health. 2009 Jun 18;8:27. doi: 10.1186/1476-069X-8-27 (PMC2708149; doi:10.1186/1476-069X-8-27)
Supplement: Additional file 7 — Characteristics of subjects (cities of Turin, Milan and Rome) included in the internal validation analysis. Reported exposure to traffic in the whole sample (first column). For the symptom of "cough or phlegm", exposure to traffic in the subgroups of subjects that could be matched by census block (in which at least one symptomatic and one asymptomatic subject were living), and in the subgroups of subjects living in census blocks where only asymptomatic subjects and only symptomatic subjects were present. [file 1476-069X-8-27-S7.doc]

**Characteristics of subjects (cities of Turin, Milan and Rome) included in the internal validation analysis.**

Reported exposure to traffic in the whole sample (first column). For the symptom of “cough or phlegm”, exposure to traffic in the subgroups of subjects that could be matched by census block (in which at least one symptomatic and one asymptomatic subject were living), and in the subgroups of subjects living in census blocks where only asymptomatic subjects and only symptomatic subjects were present.

|  |  |  | | **Whole sample**  **(n=4,210 census blocks)** | | **Subgroup matched by census block (n=498 census blocks)** | | **Subjects living in census block where only asymptomatic subjects were present (N=3,533 census blocks)** | | **Subjects living in census block where only symptomatic subjects were present (N=179 census blocks)** | |
| --- | --- | --- | --- | --- | --- | --- | --- | --- | --- | --- | --- |
| In the zone of residence | | |  |  |  |  |  |  |  |  |  |
|  | Traffic density: | |  |  |  |  |  |  |  |  |  |
|  | - absent/low |  |  | 2,708 | 30,0 | 840 | 34.3 | 1,827 | 28.5 | 41 | 21.8 |
|  | - moderate |  |  | 3,575 | 39,6 | 941 | 38.5 | 2,564 | 40.1 | 70 | 37.2 |
|  | - high |  |  | 2,592 | 28,7 | 619 | 25.3 | 1,899 | 29.7 | 74 | 39.4 |
|  | *- Missing* |  |  | *159* | 1,8 | *46* | 1.9 | 110 | 1.7 | *3* | 1.6 |
| In the street of residence | | |  |  |  |  |  |  |  |  |  |
|  | Daily lorry transit: | |  |  |  |  |  |  |  |  |  |
|  | - never |  |  | 2,666 | 29,5 | 717 | 29.3 | 1,910 | 29.8 | 39 | 20.7 |
|  | - sometime |  |  | 3,928 | 43,5 | 1,081 | 44.2 | 2,766 | 43.2 | 81 | 43.1 |
|  | - frequently |  |  | 1,791 | 19,8 | 469 | 19.2 | 1,271 | 19.9 | 51 | 27.1 |
|  | - continuously |  |  | 474 | 5,2 | 133 | 5.4 | 328 | 5.1 | 13 | 6.9 |
|  | *- Missing* |  |  | *175* | 1,9 | *46* | 1.9 | *125* | 2.0 | *4* | 2.1 |
|  | Daily cars transit: | |  |  |  |  |  |  |  |  |  |
|  | - never/sometimes |  |  | 2,386 | 26,4 | 679 | 27.8 | 1,672 | 26.1 | 35 | 18.6 |
|  | - frequently |  |  | 3,316 | 36,7 | 906 | 37.0 | 2,345 | 36.6 | 65 | 34.6 |
|  | - continuously |  |  | 3,173 | 35,1 | 816 | 33.4 | 2,274 | 35.5 | 83 | 44.1 |
|  | *- Missing* |  |  | *159* | 1,8 | *45* | 1.8 | *109* | 1.7 | *5* | 2.7 |
|  | **Total** |  |  | **9,034** | **100,0** | **2,446** | **100,0** | **6,400** | **100,0** | **188** | **100,0** |
