# Supplementary material for: Respiratory symptoms in children living near busy roads and their relationship to vehicular traffic: results of an Italian multicenter study (SIDRIA 2)
Source: Environ Health. 2009 Jun 18;8:27. doi: 10.1186/1476-069X-8-27 (PMC2708149; doi:10.1186/1476-069X-8-27)
Supplement: Additional file 8 — Associations between traffic indicators in the overall sample (n = 33,632), by source of information. SIDRIA 2. Table A: for both age groups (6–7 years and 13–14 years), information on exposure and symptoms are those reported by parents. Table B: for the younger age group (6–7 years), information on exposure and symptoms are those reported by parents, while for the older age group (13–14 years), the exposure is reported by parents and symptoms are reported by adolescents. [file 1476-069X-8-27-S8.doc]

**Associations between traffic indicators in the overall sample (n=33,632), by source of information. SIDRIA 2.**

Table A: for both age groups (6-7 years and 13-14 years), information on exposure and symptoms are those reported by parents.

|  | **Asthma symptoms** | | **Cough or phlegm** | |
| --- | --- | --- | --- | --- |
|  | **OR*** | **95% CI** | **OR*** | **95% CI** |
| **Traffic density** |  |  |  |  |
| Absent | 1.00 |  | 1.00 |  |
| Low | 1.04 | 0.93-1.16 | 0.94 | 0.80-1.10 |
| Moderate | 1.13 | 1.01-1.26 | 1.04 | 0.89-1.22 |
| High | 1.17 | 1.03-1.33 | 1.24 | 1.04-1.49 |
| **Frequency cars transit** |  |  |  |  |
| Never | 1.00 |  | 1.00 |  |
| Sometimes | 0.95 | 0.83-1.10 | 0.90 | 0.74-1.10 |
| Frequently | 1.06 | 0.93-1.22 | 1.02 | 0.84-1.26 |
| Continuously | 1.16 | 1.00-1.33 | 1.32 | 1.08-1.63 |
| **Frequency trucks transit** |  |  |  |  |
| Never | 1.00 |  | 1.00 |  |
| Sometimes | 1.10 | 1.02-1.19 | 1.14 | 1.02-1.26 |
| Frequently | 1.16 | 1.05-1.29 | 1.41 | 1.23-1.61 |
| Continuously | 1.27 | 1.08-1.50 | 1.67 | 1.36-2.06 |

*All ORs were adjusted for study centre, age, sex, parental asthma or allergies, parental education, passive smoke at home, indoor moulds, season, person filling the questionnaire, floor level of the apartment and change of residence.

Table B: for the younger age group (6-7 years), information on exposure and symptoms are those reported by parents, while for the older age group (13-14 years), the exposure is reported by parents and symptoms are reported by adolescents.

|  | **Asthma symptoms** | | **Cough or phlegm** | |
| --- | --- | --- | --- | --- |
|  | **OR*** | **95% CI** | **OR*** | **95% CI** |
| **Traffic density** |  |  |  |  |
| Absent | 1.00 |  | 1.00 |  |
| Low | 1.00 | 0.91-1.09 | 0.95 | 0.83-1.10 |
| Moderate | 1.03 | 0.94-1.13 | 1.03 | 0.89-1.19 |
| High | 1.09 | 0.97-1.22 | 1.17 | 1.00-1.38 |
| **Frequency cars transit** |  |  |  |  |
| Never | 1.00 |  | 1.00 |  |
| Sometimes | 0.97 | 0.86-1.10 | 0.96 | 0.81-1.13 |
| Frequently | 1.04 | 0.92-1.17 | 1.11 | 0.93-1.31 |
| Continuously | 1.11 | 0.98-1.25 | 1.29 | 1.08-1.55 |
| **Frequency trucks transit** |  |  |  |  |
| Never | 1.00 |  | 1.00 |  |
| Sometimes | 1.05 | 0.99-1.12 | 1.07 | 0.97-1.18 |
| Frequently | 1.09 | 0.99-1.19 | 1.29 | 1.14-1.46 |
| Continuously | 1.15 | 1.00-1.34 | 1.44 | 1.20-1.75 |

*All ORs were adjusted for study centre, age, sex, parental asthma or allergies, parental education, passive smoke at home, indoor moulds, season, person filling the questionnaire, floor level of the apartment and change of residence.
